# Supplementary material for: The Portable Microhaplotype Object and Tools
Source: bioRxiv. 2025 Dec 12:2025.12.10.693568. Preprint. [Version 1] doi: 10.64898/2025.12.10.693568 (PMC12713663; doi:10.64898/2025.12.10.693568)
Supplement: Supplement 1 [file media-1.pdf]

Supplementary Materials

Supplementary Text 1 - PMO Schema

The LinkML-based schema is kept to date on GitHub. This repository has the yaml file that defines the schema and a live page that details the structure including all fields within PMO.

GitHub (live version, continuously updated):
https://github.com/PlasmoGenEpi/portable-microhaplotype-object
Live Page:
https://plasmogenepi.github.io/portable-microhaplotype-object/
Zenodo Reference (version v1.0.0 from time of publication):
https://doi.org/10.5281/zenodo.17705710

Supplementary Text 2 - PMO Documentation

The documentation for the PMO file format is kept up to date on its own GitHub page. This contains the details on the file format as well as tutorials and example files.

GitHub (live version, continuously updated):
https://github.com/PlasmoGenEpi/PMO_Docs
Live Documentation Page:
https://plasmogenepi.github.io/PMO_Docs/
Zenodo Reference (version v1.0.0 from time of publication):
https://doi.org/10.5281/zenodo.17783443

Supplementary Text 3 - PMO Python Implementation

pmotools-python: Toolkit for working with Portable Microhaplotype Objects (PMOs)
GitHub (live version, continuously updated):
https://github.com/PlasmoGenEpi/pmotools-python
Documentation describing installation, usage, and API examples is available and kept up to date at:
Documentation (live, continuously updated):
https://plasmogenepi.github.io/PMO_Docs/pmotools-python-usages/pmotools-python.html
For reproducibility, version 1.0.0 of pmotools, used in this manuscript, has been archived at the time of publication:
DOI (frozen at publication on Zenodo): https://doi.org/10.5281/zenodo.17728203

Supplementary Text 4 - pmotools-app

pmotools-app: A simple web app built with Streamlit that helps users convert their data into the PMO format using the `pmotools` package.
GitHub (live version, continuously updated):
https://github.com/PlasmoGenEpi/pmotools-app
Live app is currently hosted here: https://pmotools.app/
For reproducibility, version 1.0.0 of pmotools-app, used in this manuscript, has been archived at the time of publication:
DOI (frozen at publication on Zenodo): https://doi.org/10.5281/zenodo.17873672

Supplementary Text 5

All example datasets are available through the associated DOI (https://doi.org/10.5281/zenodo.17823115). The archive contains **five folders**, each corresponding to one dataset:

- **Dataset1:** Public genomic surveillance data of *Plasmodium falciparum* from four countries: Eswatini, Namibia, South Africa, and Zambia.
- **ANOSPP:** Combined *Anopheles* and *Plasmodium* data.
- **mips_v_mad4hatter:** Data from the MAD4HatTeR amplicon sequencing assay and the DR23K molecular inversion probe (MIP) assay comparison.
- **E_coli:** *Escherichia coli* datasets sourced from the Sequence Read Archive (SRA).
- **S_aureus:** *Staphylococcus aureus* datasets sourced from SRA.

For **Dataset1** and **ANOSPP**, the archive includes all raw data files as well as Jupyter notebooks used to generate the PMO. Dataset1 additionally includes the notebook used to produce Figure 3. PMOs for **all five** datasets are included.

Further details about the file structure and contents are provided in the accompanying README.md file with the archived data.

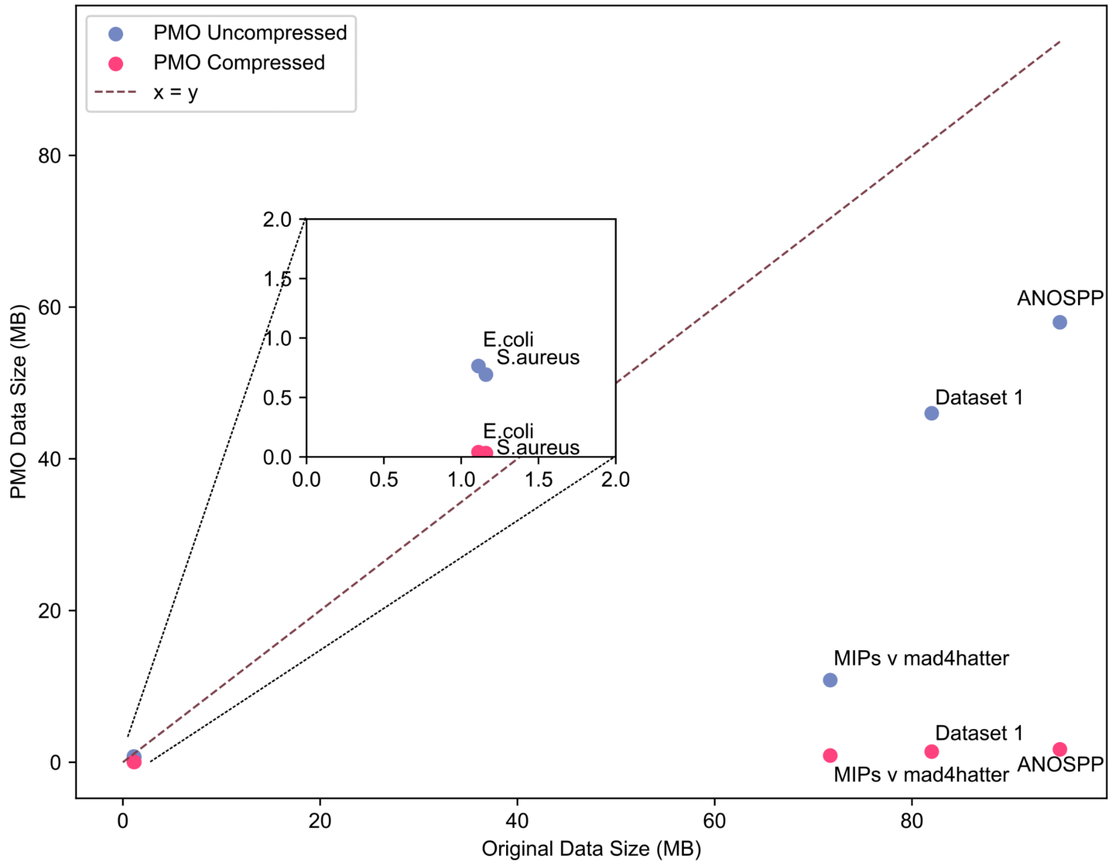

Supplementary Figure 1. Comparison of original dataset size and size after conversion to PMO. Scatter plot comparing the size of the original data (x-axis) and the corresponding PMO data (y-axis), for both uncompressed and compressed PMO outputs. All datasets show substantial reductions in size as a PMO. The inset axis provides a magnified view of the low-MB range (*S.aureus* and *E. coli*) to highlight the pronounced compression effects in small datasets.
